# Evaluation of Tumor Control and Normal Tissue Complication Probabilities in Patients Receiving Comprehensive Nodal Irradiation for Left-Sided Breast Cancer

**DOI:** 10.3390/curroncol31060241

**Published:** 2024-05-31

**Authors:** Christian H. Flores-Balcázar, Dulce M. Urías-Arce

**Affiliations:** 1 Radiotherapy and Medical Physics Service, Instituto Nacional de Ciencias Médicas y Nutrición Salvador Zubirán, Mexico City 14080, Mexico; 2 Radiotherapy Service, Instituto Nacional de Cancerología, Mexico City 14080, Mexico; dulcemaurias@gmail.com

**Keywords:** breast cancer, adjuvant radiotherapy, comprehensive nodal irradiation, radiobiological models

## Abstract

Women with left-sided breast cancer receiving adjuvant radiotherapy have increased incidence of cardiac mortality due to ischemic heart disease; to date, no threshold dose for late cardiac/pulmonary morbidity or mortality has been established. We investigated the likelihood of cardiac death and radiation pneumonitis in women with left-sided breast cancer who received comprehensive lymph node irradiation. The differences in dosimetric parameters between free-breathing (FB) and deep inspiration breath hold (DIBH) techniques were also addressed. Based on NTCP calculations, the probability of cardiac death was significantly reduced with the DIBH compared to the FB technique (*p* < 0.001). The risk of radiation pneumonitis was not clinically significant. There was no difference in coverage between FB and DIBH plans. Doses to healthy structures were significantly lower in DIBH plan than in FB plan for V20, V30, and ipsilateral total lung volume. Inspiratory gating reduces the dose absorbed by the heart without compromising the target range, thus reducing the likelihood of cardiac death.

## 1. Introduction

Breast cancer (BC) is one of the leading causes of death in women worldwide [1]. When indicated either after conservative breast surgery or after mastectomy, radiation therapy improves local control, disease-free, and overall survival by preventing local recurrence and metastatic disease [2]. Currently, the multimodality approach used to treat BC has been associated with elevated cardiac mortality since anthracycline-based chemotherapy, ERBB2 antagonists, endocrine therapy, and breast irradiation are linked to an elevated risk of lung disease, coronary artery disease, and a broad spectrum of cardiac anomalies like cardiac ischemia, heart failure, valvular heart disease, QT interval prolongation, and arrhythmias [3]. Cardiac-protective radiotherapy techniques like deep inspiration breath hold (DIBH) have evolved through time to decrease the incidence of cardiac death and coronary events, therefore allowing moderate hypofractionation and ultra-short radiotherapy schemes as a more convenient technique for patients and health institutions [4].

To date, no accurate threshold doses for late cardiac/lung morbidity or mortality have been established. The close interaction between radiobiological response models and clinical practice has yielded some evaluation tools, such as tumor control probability (TCP) and normal tissue complication probability (NTCP) models, which approximate the clinically observed treatment outcomes and complications [5]. The two best-known models are the Lyman Kutcher Burman and the relative seriality model [6,7]. The present study intended to analyze the radiobiological implication, in the form of TCP and NTCP, of the dose difference resulting from deep inspiration breath hold (DIBH) and free-breathing (FB) techniques on cardiac and lung doses during left-sided breast irradiation since the benefits of this technique as showed by radiobiological models are lacking in the medical literature.

## 2. Materials and Methods

After institutional ethical committee approval (HEM-2977-19-20-1), a sample of 22 patients referred and treated with adjuvant radiotherapy between 2015 and 2019 was randomly selected. Women aged over 18 years with left-breast-sided cancer, a histopathological diagnosis of breast carcinoma, and postoperative indication for adjuvant radiotherapy to chest wall/breast and regional nodes, including internal mammary chain, who also signed an informed consent form for both voluntary DIBH and FB planning computed tomography (CT) scan simulations were included. 

### 2.1. Simulation Procedure

As a routine procedure in the Radiation Oncology and Medical Physics Department, one week before the virtual CT simulation is performed, patients undergoing respiratory gating techniques are instructed to practice deep inspiration and breath-hold for 10 to 20 s at home. The RPM respiratory gating system (Varian Medical Systems, Palo Alto, California, USA) was used for breathing tracking. The curves generated by deep inspiration breath hold and gating window in the CT scanning were recorded and used as references for daily treatment delivery. A 16-slice CT simulation for all 22 patients was performed in the supine position using a standard breast board in free-breathing (FB) and DIBH covering the volume from the mid-neck to the upper abdomen during FB and DIBH and a slice thickness of 2.5 mm.

### 2.2. Contouring Process

For locoregional treatment after lumpectomy, the planning target volume (PTV) was the clinical limit of the breast, ipsilateral axillary lymph nodes level I–III, supraclavicular and infraclavicular fossa, and internal mammary chain. For postmastectomy radiotherapy, the PTV was defined as the part of the thoracic wall where the breast had been located and, using the contralateral breast as a reference, the ipsilateral axillary lymph nodes level I–III, supraclavicular and infraclavicular fossa, and internal mammary chain. The delineated organs at risk (OARs) were the heart, left anterior descending coronary artery (LAD), both lungs (contoured separately), thyroid, and right breast. The radiation oncologist manually delineated the heart and LAD, whereas the treatment planning system automatically delineated the lungs (Eclipse, Version 16.0, Varian Medical Systems, Palo Alto, California, USA). The heart was defined as the entire myocardium and pericardium, starting superiorly at the beginning of the pulmonary trunk and aorta. LAD was delineated from the exit of the left coronary artery from the aorta, continuing in the anterior interventricular sulcus as visualized. Regarding the treatment beams’ arrangement, identical plans were created in the DIBH and FB CT scans.

### 2.3. Radiotherapy Planning and Dosimetric Evaluation

The mono isocentric technique with supraclavicular, nondivergent tangential fields and compensating fields (field-in-field technique) was used on both CTs. We prescribed 50 Gy in 25 fractions to the mean PTV using 6, 10, or 15 MV photons. Patients with breast-conserving surgery requiring a boost to the surgical bed received a total dose of 10 Gy in 5 fractions. Dose coverage of the PTV was prioritized over the absorbed dose to the OARs and, for risk calculation, dose–volume histograms (DVHs) were revised. A TrueBeam V2.5 linear accelerator was utilized for radiation therapy and the calculation algorithm used was the Anisotropic Analytic Algorithm (AAA) version 16.1 [8].

Dosimetric goals of the treatment plans were that 95% of the PTV volume should be covered by 95% of the prescribed dose (V95%, PTV = 95%) and an acceptable value of 90% of the PTV volume should be covered by 90% of the prescribed dose (V90%, PTV = 90%). The maximum doses represented by the doses received by 2% of the target volumes (D2%), the minimum doses represented by 98% of the target volumes (D98%), and the mean doses were reported. At the same time, the absorbed dose of the OARs was kept as low as possible. The plans’ target conformity degrees were evaluated using the conformity index. The target dose homogeneity (HI) was expressed in terms of the ratio (D2%-D98%)/D50%, where D50% was the minimum dose represented by 50% of the target volumes [9]. The parameters obtained from the DVHs were mean and maximum dose to the left lung and relative volumes of the left lung receiving 5 Gy (V5), 10 Gy (V10), 20 Gy (V20), and 30 Gy (V30). Additional parameters were mean and maximum dose to the heart, relative volumes of the heart receiving at least 5 Gy (V5), 10 Gy (V10), 15 Gy (V15), 20 Gy (V20), 25 Gy (V25), 30 Gy (V30), 40 Gy (V40), and 50 Gy (V50). In addition, we also used mean and maximum dose to the LAD, and relative volumes of LAD receiving 5 Gy (V5), 10 Gy (V10), and 25 Gy (V25) [10]. 

### 2.4. Radiobiological Assessment

The software used for TCP and NTCP calculations was the Biological Evaluation Application™ V.1.6.1.4, developed by RaySearch Laboratories, Stockholm, Sweden. This software evaluates the estimated probabilities of a clinical outcome in terms of TCP Poisson-LQ for targets and NTCP Poisson-LQ for the OARs. First, each homogeneous dose level of the differential DVHs was converted to a total dose delivered in 2 Gy fractions with the biologically effective dose method, with α/β = 4.6 Gy [11]. Next, the TCP was calculated using the Poisson model for inhomogeneous dose distribution [12]. The NTCP for cardiac death risk and radiation pneumonitis were calculated using the relative seriality model [13]. Input data for the NTCP calculations with cardiac death risk as an endpoint were taken from Gagliardi et al. for the entire heart volume [14]. For radiation pneumonitis, input data were taken from the work published by Gagliardi et al. and then corrected for the AAA by the use of algorithm-specific NTCP parameters determined by Hedin et al. [15,16]. For input data see Table 1. 

### 2.5. Statistical Analysis

The Wilcoxon signed-rank test was used for continuous variables to evaluate FB vs. DIBH within each cohort. Values of *p* > 0.01 were considered statistically significant. The analysis was performed using STATA Version 18.

## 3. Results

This study analyzed several dose parameters of the target volume, left lung, heart, and LAD using two different breathing methods. All patients in our cohort could undergo the training required to use the DIBH technique. The patients’ median age in our cohort was 50 years (range 35 to 80 years), with 63.6% being postmenopausal by the time of adjuvant radiotherapy (see Table 2).

### 3.1. Plan Comparison and DVH Evaluation

All dosimetry results are shown in Table 3. There was no difference in target coverage for all patients between FB and DIBH plans. Dose to normal structures was significantly lower in DIBH plans than in the FB plans for V20 and V30. Of note is that the total ipsilateral lung volume is bigger in DIBH plans. The largest dose reductions were seen for the heart and LAD. Overall, DIBH reduced Dmean to the heart by 55.8% and the LAD by 73.3% compared to the FB plan. When DIBH was compared to FB, Dmean was 2.4 Gy versus 4.3 Gy for the heart and 6.2 Gy vs. 23.3 Gy for the LAD (*p* < 0.001), respectively. Equally significant were differences in V5, V10, V15, V20, V25, V30, V40, and V50 for the heart and V5, V10, and V25 for the LAD (*p* < 0.001).

### 3.2. TCP and NTCP Evaluation

**Figure 1:**
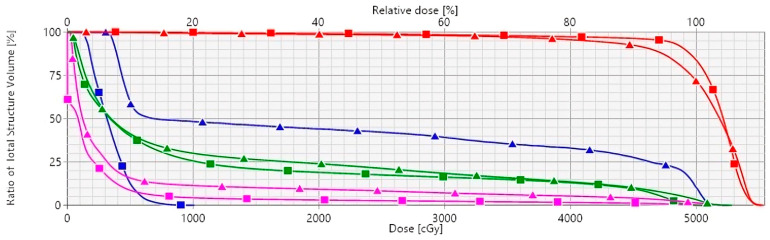
Mean dose–volume histograms comparing DIBH (square lines) and FB (triangle lines) for LAD (blue), heart (magenta), ipsilateral lung (green), and PTV (red). Abbreviations: DIBH, deep inspiration breath hold; FB, free-breathing; LAD, left anterior descending coronary artery; PTV, planning target volume.

**Figure 2:**
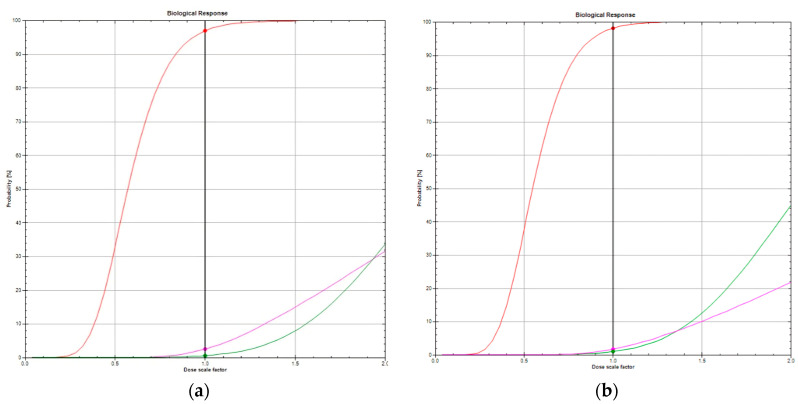
(**a**) TCP of 96.9% (red line), risk of cardiac death of 2.5% (magenta), risk of radiation pneumonitis of 0.55% (green) in a patient with FB technique. (**b**) TCP of 98.2% (red line), risk of cardiac death of 1.8% mMagenta), risk of radiation pneumonitis of 0.98% (green) in the same patient irradiated with DIBH technique. Abbreviations: DIBH, deep inspiration breath hold; FB, free-breathing; TCP, tumor control probability.

Figure 1 illustrates the DVHs for FB and DIBH radiation techniques. Figure 2a,b compare the TCPs and NTCPs for target volume and healthy tissues, respectively, among techniques. The TCP values for the chest wall/breast and supraclavicular, axillary, and internal mammary nodes were 91.2% in FB and 93.5% in DIBH (*p* = 0.006). Based on NTCP calculations, the risk of cardiac death was significantly decreased for DIBH compared to FB (1 vs. 0.45), suggesting that using DIBH for patients with left-sided breast cancer and locoregional treatment is beneficial (*p* < 0.001). The risk of radiation pneumonitis did not achieve clinical significance for FB and DIBH techniques (1.2 vs. 1.1, *p* = 0.237; Table 4).

## 4. Discussion

Breast cancer is one of the most frequent tumors in women worldwide. Even when survival for patients with this disease is longer due in part to the use of novel systemic drugs, advances in surgical approaches, and novel radiotherapy techniques, physicians are now dealing with the long-term effects of cancer treatments for which they have to be aware [16]. Physicians caring for cancer survivors have expressed concern over the potential heart and lung toxicities caused by breast radiation therapy, in addition to the side effects of chemotherapy and endocrine therapy. The Early Breast Cancer Trialists’ Collaborative Group (EBCTCG) published one of the earliest reports on the cardiopulmonary toxicity associated with breast radiotherapy in a meta-analysis. The report demonstrated a hazard ratio of 1.27 for heart disease, although it included studies that used outdated radiation techniques [17]. Women with left-breast cancer receiving radiotherapy have an increased incidence of major coronary events compared to women with right-sided cancer due to ischemic heart disease, with 7.4% per Gy of radiation received to the heart [18]. In addition to primarily mild side effects, including erythema, edema, and inflammation, that happen in the short- and mid-term, clinicians must be aware of long-term effects on the heart and lungs since most patients with BC reach long survival rates, thus embracing the long-term consequences of systemic therapies and radiotherapy [19]. Another adverse effect contributing to decreased quality of life in patients receiving thoracic radiotherapy is radiation-induced lung toxicity ultimately causing lung fibrosis [20]. The CANTO-RT trial was a prospective longitudinal cohort that recorded adverse events in patients receiving breast radiotherapy. With a 60-month follow-up, the incidence of radiation-induced lung toxicity was about 2.4% for the entire group, with pulmonary medical history, chemotherapy use, and nodal irradiation being risk factors for its occurrence [21].

Since then, much has been done to lower the risk of cardiac and lung toxicity in patients receiving radiotherapy for left-breast cancer, and the breath hold technique is one of the available methods developed to lessen this risk. A recent study published by Mahmoud et al. compared the incidence of cardiac events in patients with breast cancer who received FB (free-breathing) vs. DIBH (deep inspiration breath hold) techniques in a large retrospective cohort. Although the incidence of cardiac events was higher in the FB arm than in the DIBH arm, it did not reach statistical significance. However, the study found that having hypertension, smoking, and a high heart mean dose were independent risk factors for the occurrence of cardiac events [22]. Our study evaluated the dosimetric parameters of DIBH compared to the FB RT technique, finding a notable reduction in heart and lung volumes for DIBH. Therefore, we showed that DIBH not only reduces the Dmean and Dmax to the heart and LAD but also the risk of lung and heart toxicity using radiobiological models.

TCP and NTCP models have been developed to determine the success rate of a given RT treatment while minimizing the risks of tissue toxicity. These models combine clinical outcomes with dosimetric information regarding dose–volume histograms (DVHs). Mathematical calculations are used to derive model parameters that factor in clinical outcomes to estimate the risk of tumor relapse or toxicity. Both models condense all patient dosimetric data into DVHs, which may limit their descriptive and predictive power [23]. The study conducted by Utehina et al. highlights the potential benefits of using respiratory-gated techniques in postoperative radiation therapy for early-stage left-sided breast cancer. The results indicate that the use of respiratory-gated techniques significantly reduces the risks of pneumonitis and cardiac mortality as compared to the control group. This is a significant finding that could have a positive impact on the clinical management of breast cancer patients. The risks of pneumonitis and cardiac mortality were reduced from 0.6% to 0.3% and from 1.3% to 0.2%, respectively. This study underscores the importance of respiratory-gated techniques in reducing the risk of side effects in patients undergoing radiation therapy for breast cancer [24].

This research delves into the most suitable radiation therapy techniques for treating left-sided breast cancer patients undergoing nodal irradiation. This study evaluated two techniques frequently employed in radiation therapy and provided valuable insights. By analyzing the differences in doses and volumes of the heart and lungs during radiation therapy, both radiation techniques helped to identify the most suitable breathing technique. This study’s key finding is that the TCPs and NTCPs varied between the two techniques, with the TCP of the DIBH being higher than the FB plans (2.3%). The risk of cardiac mortality is significantly reduced when DIBH is used. This research shows that DIBH is the preferred technique for patients with left-sided breast cancer when regional nodal irradiation is necessary, as it reduces the median cardiac death risk by 0.55 percentage points. The results of this study are significant, as they guide physicians and medical practitioners on the most suitable RT techniques for treating patients with left-sided breast cancer undergoing nodal irradiation. However, it is essential to note that the retrospective nature of this publication and its sample size may be two of the main limitations, suggesting the need for further investigation in this area.

Biological models can be valuable in clinical applications as they can predict radiation response in patients. However, these predictions have some limitations due to the uncertainty of the model parameters involved. NTCP models are often used in comparative planning studies, as they provide a correct qualitative description of the radiation response, and only the ranking of NTCP values is considered. However, this type of application has a significant drawback: the uncertainty of the predictions about the model parameters is not specified quantitatively, raising questions about the significance of differences in NTCP values for different treatment plans or techniques. TCP models face similar limitations, as parameters such as proliferation, oxygenation, and angiogenesis are much more varied for tumors than for normal tissues, and may change during radiotherapy, making the clinical application of TCP models challenging. Nonetheless, the models can still help improve the understanding of tumor response to radiation and its interaction with other influencing factors [25,26].

## 5. Conclusions

The enhanced inspiration-gating technique, a significant advancement in our research, has proven to significantly decrease the absorbed radiotherapy dose to the heart and LAD coronary artery without compromising the target coverage. This success story has resulted in a decreased cardiac mortality probability, a promising outcome. However, the reduction of the risk of pneumonitis between FB and DIBH could not be demonstrated. Several biological models have been developed. Although these models correctly describe the main characteristics of the radiation response, great caution must be taken if these models are to be applied to patients.

## Figures and Tables

**Table 1:** Input parameters used in the biological evaluation application software.

Radiobiological Evaluation	Dosimetric Parameters		
Tumor Control Probability [13,14,15]	D50 *	γ **	s ***
Adjuvant pT1-pT2	39.89 Gy	1.3%/%	-
Adjuvant pT3	42.46 Gy	0.53%/%	-
Adjuvant pN1	24.55 Gy	1.07%/%	-
Adjuvant pN2	43.40 Gy	0.68%/%	-
Adjuvant pN3	50.03 Gy	2.63%/%	-
Normal Tissue Control Probability for Cardiac Death Risk [13,14]			
Entire Heart Volume	52.3 Gy	1.28%/%	1
Normal Tissue Control Probability for Radiation Pneumonitis [15,16]			
Whole Left Lung	29.23 Gy	0.966%/%	0.012

* D50 is the dose delivered to the whole organ to induce TCP or NTCP = 50%, ** γ is the dose/response steepness index, *** s represents the degree of seriality modeled for the organ.

**Table 2:** Baseline demographics for left-sided breast cancer patients in the study.

Patient and Treatment Characteristics	Distribution and %
Median Age (years), range	50 (35–80)
Hormonal Status Premenopausal Postmenopausal	8 (36.4) 14 (63.6)
AJCC Stage IIA IIB IIIA IIIB IIIC	6 (27.3) 8 (36.4) 4 (18.2) 2 (9.1) 2 (9.1)
Nodal Status Nx N0 N1 N2 N3	1 (4.6) 4 (18.6) 13 (58.6) 1 (4.6) 3 (13.6)
Tumor Location within the Left Breast Upper Inner Quadrant Lower Inner Quadrant Upper Outer Quadrant Lower Outer Quadrant	8 (36.4) 9 (40.9) 3 (13.6) 2 (9.1)
Molecular Classification Luminal A Luminal B Pure Her2 Triple Negative	5 (22.7) 9 (40.9) 3 (13.6) 5 (22.7)
Histology Ductal Invasive Lobular Invasive	20 (90.9) 2 (9.1)
Grade 1 2 3 N/R	2 (9.1) 9 (40.9) 9 (40.9) 2 (9.1)
Type of Surgery Breast-Conserving + Sentinel Node Procedure Mastectomy + Axillary Dissection	9 (40.9) 13 (59.1)
Radiation Treatment Fields Breast + Supraclavicular + Internal Mammary Chest Wall + Supraclavicular + Internal Mammary	9 (40.9) 13 (59.1)
Timing of Chemotherapy Neoadjuvant Adjuvant	12 (54.5) 10 (45.5)
Systemic Treatment Doxorubicin + Cyclophosphamide followed by Paclitaxel Docetaxel + Cyclophosphamide Capecitabine	19 (86.4) 2 (9.1) 1 (4.6)
Adjuvant Endocrine Therapy Yes No	14 (63.6) 8 (36.4)

Abbreviations: AJCC, American Joint Committee on Cancer; N/R, not recorded.

**Table 3:** Treatment planning data for target and organs at risk for FB and enhanced DIBH for locoregional treatment presented as median values with standard deviation and *p*-values for paired Wilcoxon tests.

Parameters	FB (Median and SD)	DIBH (Median and SD)	*p*-Value
Target Volume V95 (%) V105 (%) V107 (%) D2 (Gy) D98 (Gy) Dmean (Gy) CI HI	89.0 ± 5.9 35.1 ± 13.7 14.2 ± 11.2 54.5 ± 0.5 41.1 ± 4.2 50.7 ± 0.8 0.7 ± 0.1 0.2 ± 0.1	90.3 ± 4.1 38.0 ± 14.5 18.8 ± 12.9 54.5 ± 0.8 41.6 ± 4.5 51.0 ± 0.7 0.8 ± 0.1 0.2 ± 0.1	0.390 0.115 0.217 0.961 0.417 0.026 0.198 0.794
Left Lung Dose V5 (%) V10 (%) V20 (%) V30 (%) Dmean (Gy) Dmax (Gy) Total Volume	43.9 ± 9.1 30.3 ± 7.5 23.6 ± 6.5 19.8 ± 5.9 12.5± 8.7 52.5 ± 1.3 1133.7 ± 240.1	43.1 ±7.6 28.5 ± 6.0 21.1 ± 4.9 17.8 ± 4.1 12.0 ± 2.3 52.4 ± 1.2 1919.2 ± 364.4	0.638 0.095 0.036 0.016 0.067 0.130 **<0.001**
Heart V5 (%) V10 (%) V15 (%) V20 (%) V25 (%) V30 (%) V40 (%) V50 (%) Dmean (Gy) Dmax (Gy)	14.3 ± 6.1 9.4 ± 5.3 8.3 ± 4.9 6.8 ± 4.8 5.8 ± 4.6 5.3 ± 4.4 3.9 ± 3.9 0.4 ± 1.4 4.3 ± 2.3 51.8 ± 1.6	6.6 ± 4.7 3.8 ± 3.4 2.8 ± 2.9 2.2 ± 2.6 2.2 ± 2.4 1.9 ± 2.0 1.2 ± 1.4 0 ± 0.3 2.4 ± 1.2 50.3 ± 10.4	**<0.001** **<0.001** **<0.001** **<0.001** **<0.001** **<0.001** 0.003 **<0.001** **<0.001** **<0.001**
LAD V5 (%) V10 (%) V25 (%) Dmean (Gy) Dmax (Gy)	80.6 ± 26.8 60.6 ± 34.6 43.1 ± 33.3 23.2 ± 13.3 49.5 ± 13.1	56.5 ± 26.1 8.6 ± 25.8 0 ± 22.9 6.2 ± 8.4 24.5 ± 15.8	0.014 **<0.001** **<0.001** **<0.001** **<0.001**

Abbreviations: CI, conformity index; D2, dose registered in 2% of the planning target volume; D98, dose registered in 98% of the planning target volume; DIBH, deep inspiration breath hold; FB, free-breathing; HI, homogeneity index; LAD, left anterior descending coronary artery; V10, percentage of volume receiving 10 Gy; V105, percentage of volume receiving 105% of prescribed dose; V107, percentage of volume receiving 107% of prescribed dose; V15, percentage of volume receiving 15 Gy; V20, percentage of volume receiving 20 Gy; V25, percentage of volume receiving 25 Gy; V30, percentage of volume receiving 30 Gy; V40, percentage of volume receiving 40 Gy; V5, percentage of volume receiving 5 Gy; V50, percentage of volume receiving 50 Gy; V95, percentage of volume receiving 95% of prescribed dose.

**Table 4:** Tumor control probability, cardiac death risk, risk of radiation pneumonitis in percent for locoregional treatment, presented as median values, range in brackets and *p*-values for paired Wilcoxon tests.

Parameters	FB	DIBH	*p*-Value
Tumor Control Probability	91.5 [7.8–98.3]	93.5 [20.9–98.3]	0.006
Cardiac Death Risk	1.0 [0.1–5.7]	0.45 [0–2.4]	**<0.001**
Risk of Radiation Pneumonitis	1.2 [0–4.6]	1.1 [0–3.9]	0.237

Abbreviations: FB, free-breathing; DIBH, deep inspiration breath hold.

## Data Availability

The corresponding author can provide the database to the reader upon request.
